# Sodium-Glucose Cotransporter 2 Inhibitor (SGLT2i) Associated Diabetic Ketoacidosis in Oncology Patients: A Case Series and Literature Review

**DOI:** 10.7759/cureus.53816

**Published:** 2024-02-07

**Authors:** Fatima AlKindi, Yousef Boobes, Fatima Shalwani, Jawaher Ansari, Raya Almazrouei

**Affiliations:** 1 Department of Internal Medicine, Tawam Hospital, Al Ain, ARE; 2 Seha Kidney Care, Tawam Hospital, Al Ain, ARE; 3 Department of Internal Medicine, College of Medicine and Health Sciences, Al Ain, ARE; 4 Department of Oncology, Tawam Hospital, Al Ain, ARE; 5 Division of Endocrinology, Tawam Hospital, Al Ain, ARE

**Keywords:** oncology, chemotherapy, diabetes mellitus type 2, dka, sglt2i

## Abstract

Sodium-glucose cotransporter 2 inhibitors (SGLT2i) use is associated with an increased risk of diabetic ketoacidosis (DKA). The clinical data regarding the use of SGLT2i and its potential side effects in oncology patients is limited. We are retrospectively reporting four oncology patients with type 2 diabetes mellitus using SGLT2i who were admitted with DKA. The mean age of the patients was 61.25 years, and male to female ratio was 1:1. The duration of type 2 diabetes ranged from 10 to 20 years (mean 15.75 years) and the types of SGLT2i used were empagliflozin 25 mg and dapagliflozin 10 mg. The types of malignancy in our case series included squamous cell carcinoma of the cheek, ovarian cancer, and two patients had laryngeal carcinoma (squamous cell carcinoma). Diabetic ketoacidosis was diagnosed in three patients following chemotherapy or concurrent chemo-radiotherapy. Poor oral intake and infections were the main risk factors in our patients. Mean blood glucose level, anion gap, and bicarbonate level were 11.7 mmol/l, 32.25, and 5 mmol/l, respectively. The majority had moderate DKA based on pH (mean 7.13). The hospital course was complicated by acute kidney injury (n=4), infections (n=4) (urinary tract infections, and pneumonia), and three patients required critical care. The mean length of hospitalization was 19.2 days and no mortality was reported among our patients. SGLT2i-related DKA is an emerging complication recognized in oncology patients. Some of the risk factors for this complication are starvation, poor oral intake, and infection which are quite prevalent in oncology patients. Temporary holding of SGLT2i medication during this period might have a potential preventive role.

## Introduction

Sodium-glucose cotransporter 2 inhibitors (SGLT2i) is a novel class of hypoglycemic medications approved by the Food and Drug Administration (FDA) in 2013 for glycemic control in Type 2 diabetes (T2DM) patients. Several randomized control studies showed the beneficial effects of SGLT2i in the management of diabetic kidney disease, heart failure (reduced and preserved ejection fraction) and reduction of cardiovascular events and all-cause mortality [1].

Diabetic ketoacidosis (DKA) is a potentially life-threatening complication that occurs in Type 1 diabetes (T1DM) patients more than T2DM. The diagnostic criteria for DKA include blood glucose level > 250 mg/dL (13.9 nmol/L), positive ketones tests, and metabolic acidosis (serum bicarbonate level < 15 mEq/L, high anion gap metabolic acidosis, arterial pH < 7.3) [2]. In euglycemic DKA, the blood glucose level is below 250 mg/dL (13.9 nmol/L). Noncompliance to diabetic medications, severe infection, new diagnosis of diabetes, critical condition, and starvation are potential risk factors for DKA [3]. In 2015, the FDA issued a warning about SGLT2i-induced DKA in patients with diabetes. In fact, the use of SGLT2i in patients with T2DM diabetes has been associated with an increased risk of DKA, ranging from two to sevenfolds [4-6]. Risk factors predisposing to DKA with SGLT2i use include poor oral intake, surgery, alcohol, and infection [6,7]. A recent study published by our center showed that 31% (17/55) of patients with T2DM admitted with DKA were using SGLT2i and it is use was associated with a fivefold increased risk of prolonged hospital stay (>=14 days) [7]. The diagnosis of SGLT2i-induced DKA may be delayed due to relatively normal blood glucose at presentation in many patients. SGLT2i-induced DKA in oncology patients with diabetes is rarely reported. This case series aims to describe the clinical characteristics and management outcomes of SGLT2i induced DKA in four oncology patients.

## Case presentation

Case 1

A 65-year-old woman presented to the emergency department (ED) with a three-day history of severe mucositis and poor oral intake after cycle 4 of chemotherapy. She was diagnosed with moderately differentiated squamous cell carcinoma (Stage: cT4aN2M0) in the right inner cheek and was initiated on concurrent chemotherapy (cetuximab) and radiotherapy. Additionally, she was known to have hypertension (HTN), T2DM and diabetic nephropathy, and status post-kidney transplantation. Her home medications included insulin glargine, dapagliflozin 10 mg, pantoprazole 40 mg, prednisolone 7.5 mg daily, and cyclosporine 125 mg twice daily. At presentation, she was in moderate distress, afebrile with sinus tachycardia of 117 beats per minute (bpm) and blood pressure (BP) of 135/62 mmHg. Physical examination was significant for severe oral mucositis with ulcerations and radiation dermatitis in the right facial area. Laboratory investigations revealed a blood glucose level of 12 mmol/L (3.9-7.8 mmol/L), hyperkalemia of 5.7 mmol/L (3.2-5.5 mmol/L), high anion gap of 33 mmol/L (10 mmol/L), bicarbonate level of 6 mmol/L (22-29 mmol/L) and venous pH of 7.1 (7.35-7.45). Additionally, she had evidence of acute kidney injury (AKI) stage 1 in the transplanted kidney (serum creatinine increased from a baseline of 58 to 110 micromole/L (44-80 micromole/L) and urine was positive (severe) for ketones. She was diagnosed with SGLT2i (dapagliflozin 10 mg) related euglycemic DKA with poor oral intake and starvation as provoking factors. She required critical care admission with rigorous intravenous (IV) hydration, continuous insulin infusion per DKA protocol, IV sodium bicarbonate, IV hydrocortisone, and IV cyclosporine. Her clinical condition improved by the fifth day of admission. The hospital stay length was 15 days and was complicated by an episode of urinary tract infection, nosocomial pneumonia, and re-feeding syndrome post nasogastric enteral feeding. SGLT2i was held and not given after discharge.

Case 2

A 64-year-old man presented to ED with fever, nausea, repeated vomiting, abdominal pain, cough, dyspnea, and dysuria that started three days post-discharge. He was diagnosed with laryngeal squamous cell carcinoma (TNM stage pT4a, N0, Mx) and underwent laryngectomy with total radical neck dissection and tracheostomy creation in February 2017. He was known to have uncontrolled T2DM (HbA1c 10.1%), HTN, and dyslipidemia (DLP). His home medications included amlodipine 10 mg, rosuvastatin 10 mg, gliclazide 60 mg, linagliptin-metformin 2.5-1000 mg twice daily, and empagliflozin 25 mg that was started on day 9 post-operatively prior to home discharge. At presentation, he was febrile (38.5 °C) with tachycardia of 113 bpm, tachypneic with 25 breath per minute (Bpm) on 6 L/min O_2_ via tracheostomy and hypotensive with BP of 89/55 mmHg. Investigations revealed leukocytosis 17.4 x10^9^/L (4.5-11 x10^9^/L), anemia 113 g/L (11.7-16.1 g/L), and severe thrombocytopenia 19 x10^9^/L (140-400 x10^9^/L). In addition, he had hyponatremia of 129 mmol/L, hyperglycemia 14.1 mmol/L, high anion gap of 24 mmol/L, bicarbonate level of 16 mmol/L and pH of 7.28. He had pre-renal acute kidney injury stage II with creatinine increase from a baseline of 36 micromole/L to 129 micromole/L and urine was positive (severe) for ketones. He was diagnosed with SGLT2i (empagliflozin 25 mg) related DKA with sepsis (Enterobacter bacteremia with trachea-bronchitis and UTI) and poor oral intake as provoking factors. He was admitted to the intensive care unit (ICU). Intravenous hydration, IV antibiotics (cefepime, clindamycin) and continuous insulin infusion per DKA protocol were initiated. He developed severe thrombocytopenia and autoimmune hemolytic anemia managed with IV immunoglobulin and prednisolone 1 mg/kg for two weeks. The duration of hospitalization was 16 days. The SGLT2i was not resumed after discharge from the hospital. He received chemotherapy and radiotherapy during his oncology follow-up visits. 

Case 3

A 59-year-old man presented to ED with a 10-day history of fatigue, poor oral intake, neck pain, mouth mucositis, ulceration post-radiation, and reduced urine output. He was diagnosed with laryngeal carcinoma stage cT3N0M0 and started concurrent chemo (weekly cisplatin) and radiotherapy (70 Gy/35 fractions to the larynx and 45 Gy to B/L neck) in May 2021. He was known to have T2DM, HTN, DLP, and ischemic heart disease (IHD). His medications included dapagliflozin 10 mg, gliclazide SR 30 mg twice daily, lisinopril 10 mg, atorvastatin 20 mg, and aspirin 100 mg. He continued to take his medications despite poor oral intake. At presentation, he was afebrile at 37.3 °C with sinus tachycardia at 133 bpm, tachypnea at 22 Bpm, and BP of 90/70 mmHg. Apart from dehydration, he had diffused mouth mucositis with ulceration and radiation dermatitis in the left neck area. Laboratory investigations showed a serum glucose level of 10.5 mmol/L, hyperkalemia of 5.8 mmol/ L, high anion gap of 26 mmol/L, bicarbonate level of 7 mmol/L, and PH of 7.08. Additionally, he had AKI (creatinine increased from baseline of 53 micromole/L to 122 micromole/L) and POCT (point of care test) ketones of 6.3 mmol/L along with positive (moderate) urine ketones. He was diagnosed with SGLT2i (dapagliflozin) related euglycemic DKA with poor oral intake and starvation as provoking factors. He was admitted for the management of DKA and radiation-induced mucositis. Intravenous hydration, and insulin therapy per DKA protocol were initiated. Later, feeding via nasogastric tube was started after which he developed an episode of re-feeding syndrome. His clinical condition improved, and was discharged after seven days. The SGLT2i medication was not resumed. 

Case 4

A 53-year-old woman presented to ED with a one-week history of poor oral intake, vomiting, fever, abdominal pain, and dysuria after her first cycle of palliative chemotherapy (doxorubicin/bevacizumab). She was diagnosed with ovarian carcinoma in 2016 and underwent a total abdominal hysterectomy and bilateral oophorectomy. She received several lines of chemotherapy, however, in June 2020, she developed metastatic disease with recurrent ascites for which palliative chemotherapy was initialed. Also, she is a known case of T2DM on empagliflozin 25 mg. At presentation, she was afebrile with sinus tachycardia of 117 bpm, tachypnea of 24 Bpm, and blood pressure of 118/75 mmHg. Physical examination was remarkable for tender abdominal ascites. Laboratory investigations showed a serum blood glucose level of 11.9 mmol/ L, a high anion gap of 26 mmol/L, a bicarbonate level of 5 mmol/l and a pH of 7.08. Additionally, she developed AKI (Creatinine increased from a baseline of 36 micromole/L to 111 micromole/L) and positive (moderate) urine ketones. Urinalysis showed leukocyte esterase, elevated urine WBC 87 x10^6^/L (<10 x10^6^/L), and urine RBC 99 x10^6^/L (<5 x10^6^/L). She was diagnosed with SGLT2i (empagliflozin) related euglycemic DKA with poor oral intake, starvation, and urinary tract infection as provoking factors. She was managed with intravenous hydration, antibiotics, and continuous insulin infusion as per DKA protocol. Her hospital course was complicated by refractory symptomatic malignant ascites with abdominal compartment syndrome and repeated vomiting. She had partial improvement of symptoms with drainage of ascites. She had a prolonged hospital stay of 31 days.

All the above cases are summarized in Table 1.

**Table 1:** Clinical characteristics of the four oncology patients with SGLT2i-induced diabetic ketoacidosis (DKA) T2DM; type 2 diabetes mellitus, HTN; hypertension, DLP; dyslipidemia, IHD; ischemic heart disease, AG; anion gap, UTI; urinary tract infection, ICU; intensive care unit, NGT; nasogastric tube.

Variables	Case 1	Case 2	Case 3	Case 4
Age/sex	65/F	64/M	59/M	53/F
Comorbid conditions	T2DM, HTN, Status post kidney transplantation	T2DM, HTN, DLP	T2DM, HTN, DLP, IHD	T2DM
Duration of T2DM	20 years	10 years	> 20 years	13 years
Type of SGLT2i	Dapagliflozin 10 mg	Empagliflozin 25 mg	Dapagliflozin 10mg	Empagliflozin 25 mg
Type of cancer	Inner cheek squamous cell carcinoma (stage cT4aN2M0)	Laryngeal squamous cell carcinoma (stage pT4a, N0, Mx)	Laryngeal carcinoma (stage c T3N0M0)	Metastatic Ovarian Carcinoma
Oncology Treatment at the time of DKA	Concurrent chemotherapy (Cetuximab) and radiotherapy	Post operation, not started on chemo or radiotherapy	Concurrent chemotherapy (Cisplatin) and radiotherapy	Palliative chemotherapy (doxorubicin/bevacizumab)
Initial labs:				
PH (unitless)	7.1	7.28	7.08	7.08
Glucose (mmol/L)	12	14.1	11	11.9
Bicarbonate (mmol/L)	6	16	7	5
Potassium (mmol/L)	5.7	4.4	5.4	3.3
Anion gap (mmol/L)	33	24	26	46
Urinary Ketones	Positive	Positive	Positive	Positive
Risk factors for DKA	Poor oral intake & severe mucositis	Poor oral intake, Infection (UTI) & bronchitis	Poor oral intake & severe mucositis	Poor oral intake & UTI
Treatment provided:				
IV hydration	Yes	Yes	Yes	Yes
Insulin Infusion	Yes	Yes	Yes	Yes
IV sodium bicarb	Yes	No	No	No
Need for ICU	Yes	Yes	No	Yes
Acute Kidney Injury	Yes	Yes	Yes	Yes
Other Complications	UTI, Nosocomial pneumonia & re-feeding syndrome	Autoimmune hemolysis and ITP	Pancytopenia, re-feeding syndrome	Malignant ascites & abdominal compartment syndrome
Hospital stay (days) mortality	15	16	7	31
Death during hospitalization	No	No	No	No

## Discussion

We described four oncology patients with T2DM who developed DKA secondary to continuous SGLT2i use despite starvation and poor oral intake. Diabetic ketoacidosis was diagnosed in three patients following chemotherapy or concurrent chemo-radiotherapy. The types of malignancy in our case series included squamous cell carcinoma of the cheek, laryngeal carcinoma, and ovarian cancer. The clinical condition in the reported cases improved with IV hydration and insulin infusion and was associated with prolonged hospital stay.

Diabetes mellitus can be a comorbid disease, or it can develop after diagnosis of cancer. In a population cohort study, it was found that cancer is a risk factor for developing diabetes mellitus with the hazard ratio (HR) of 1.35 (95% CI, 1.26-1.45; p<.001) and the highest risk was observed during the first two years after diagnosis of pancreatic, kidney, liver, breast, and thyroid cancers [8]. This has occurred possibly due to the use of chemotherapy agents such as hormonal therapy and corticosteroids. 

SGLT2i use is associated with reduced all-cause mortality with lower risk of cardiac events (heart failure, ischemic heart disease) and chronic kidney disease [1]. In the oncology field, it has been found that SGLT2 inhibitor use resulted in reduced mortality (HR = 0.68, 95% CI = 0.60-0.77) in diabetic patients with non-small cell lung cancer [9] and improved overall survival in diabetic patients with hepatocellular carcinoma (HCC) [10]. However, SGLT2 inhibitors are associated with an increased risk of mycotic genital infections, acute kidney injury, dehydration, and euglycemic DKA. Several precipitating risk factors may increase the risk of DKA among SGLT2 inhibitors including prolonged starvation, acute illness (infection, myocardial infarction), alcohol intoxication, and surgical procedures [11]. Euglycemic DKA was reported in all types of SGLT2i and was frequently observed with canagliflozin followed by dapagliflozin and empagliflozin [12].

There are a few cases in the literature reporting SGLT2i-related DKA in oncology patients that are summarized in Table 2 [13-17]. This was observed with the use of dapagliflozin and empagliflozin as they are the widely used types of SGLT2i with additional approval use for heart failure and chronic kidney disease (CKD). Two patients had adenocarcinoma of the pancreas [15,16], two patients with lung cancer [13,17], and one with colon cancer [14]. The majority of the reported cases developed DKA prior to the initiation of cancer-specific therapy.

**Table 2:** Summary of the reported cases of SGLT2i-induced DKA in oncology patients T2DM; type 2 diabetes mellitus, HTN; hypertension, CVD; cerebrovascular disease, AG; anion gap, ICU; intensive care unit, DKA; Diabetic ketoacidosis.

Cases	Sexe et al., 2020 [13]	Papadokostaki and Liberopoulos, 2019 [14]	Sezer et al., 2020 [15]	Luo et al., 2022 [16]	O’Neill et al., 2020 [17]
Age/Sex	58/M	64/M	58/M	57/F	71/M
Comorbid conditions	T2DM, HTN	T2DM, HTN	T2DM	T2DM, HTN, CVD (stroke)	T2DM, previously treated bladder carcinoma with ureteric ileostomy
SGLT2i type	Empagliflozin 10 mg	Dapagliflozin 10 mg	Empagliflozin 10 mg	Dapagliflozin 5 mg	Empagliflozin 10 mg
Type of cancer	Lung adenocarcinoma (stage IV)	Colon cancer underwent left hemicolectomy	Poorly differentiated adenocarcinoma of the pancreas	Poorly differentiated pancreatic ductal adenocarcinoma	Non-small-cell lung metastatic adenocarcinoma (stage IV)
Oncology treatment at the time of DKA	One day of chemotherapy (no available details). Completed five weeks of radiation therapy	No therapy, he was diagnosed at the same time as DKA	No therapy, he was diagnosed at the same time as DKA	Laparoscopic distal pancreatectomy with en bloc splenectomy. On somatostatin	Not started on cancer therapy yet
Initial labs:					
pH (unitless)	-	7.33	7.07	7.09	7.343
Glucose (mmol/L)	6.3	11.3	11.4	12.4	5.1
Bicarbonate (mmol/L)	13	10.9	2.7	14	13.6
Ketones	Positive in urine	Positive in urine	Serum 3.5 mmol/L	Serum 10.87 mmol/L	Serum 7.4 mmol/L
Potassium (mmol/L)	4.4	4.6	3.9	-	-
Anion gap (mmol/L)	-	29	-	36.2	19.9
Treatment					
IV hydration	Yes	Yes	Yes	Yes	Yes
Insulin Infusion	Yes at 6.9 U/h	Yes	Yes	Yes	Yes
IV sodium bicarb	No	No	No	No	No
Need for ICU	Yes	-	-	Yes	Yes
Other Complications	Neutropenia	No complications	No complications	Shock and Intubated	Pulmonary embolism and hospital-acquired pneumonia
Hospital stay (days) mortality	6	9	-	42	15
Death during hospitalization	No	No	No	No	No

Some studies reported longer hospital stays with SGLT2i-related DKA episodes compared to non-users [7,18], while another study showed no difference [19]. In our previous study [7], we proposed that the longer hospital stay with the SGLT2i use could be multifactorial. Delay in the diagnosis due to relatively normal blood glucose, lack of knowledge about this risk among other health care specialties, worse severity of DKA at presentation, hypovolemia, and the higher incidence of AKI are possible reasons for prolonged hospital admission. In oncology patients, other cancer-specific therapy complications could also lead to prolonged hospitalization in such cases.

In addition, several cases have been reported regarding prolonged courses of SGLT2i-induced DKA that required higher insulin doses along with additional measures to reverse the persistent ketonemia [20,21]. While glucose is used for insulin titration in DKA protocol in some centers, others use POCT ketones to titrate the insulin infusion rate. Though most SGLT2i-associated DKA can be successfully managed with standard DKA protocol, it is worth exploring whether insulin titration based on glucose vs POCT ketones would be a better option in this case.

It is important to adhere to the preventive strategies that can mitigate the risk of DKA with SGLT2i use. These strategies include proper patient selection for SGLT2i use, excluding T1DM and those on ketogenic diets [22]. Additionally, patients need to be educated regarding holding the drug during illness with poor oral intake or dehydration with early presentation to ED in case of any symptoms suggestive of DKA. Since many oncology patients under active cancer-specific therapy may suffer from poor oral intake, starvation, and gastrointestinal side effects, it is worth ensuring that the patients hold the drug during this time. Adding to that, it is worth expanding awareness among other healthcare providers about the necessity to suspend these drugs during hospital admissions or preoperatively [23].

## Conclusions

SGLT2i-related DKA is an emerging complication that is worth being aware of with the expanding list of indications for SGLT2i use. Oncology patients with risk factors like starvation, poor oral intake, surgery, and infection are at risk. Temporary holding of SGLT2i medication during this period might have a potential preventive role. In addition, education to both patients and healthcare professionals about this risk and its preventive strategies is very crucial.
